# Rehabilitation in severe memory deficit: A case study

**DOI:** 10.1590/1980-57642016dn11-020016

**Published:** 2017

**Authors:** Nariana Mattos Figueiredo Sousa

**Affiliations:** 1 Neuropsicóloga. Psicóloga Hospitalar do Programa de Reabilitação Neurológica da Unidade Salvador da Rede Sarah de Hospitais de Reabilitação

**Keywords:** amnesia, neuropsychological tests, rehabilitation, amnésia, testes neuropsicológicos, reabilitação

## Abstract

The term amnesia refers to a pathological state of mind in which memory and learning are affected to a greater extent than other cognitive functions in a patient without altered level of consciousness. The aim of the current study was to describe a case of severe amnesia in a patient during neurological rehabilitation and to report the importance of preserved cognitive functions to compensate for the mnemonic deficit. VJA presented a clinical condition suggestive of encephalopathy due to caloric-protein malnutrition following several abdominal surgical procedures for complicated choledocholithiasis. A descriptive analysis of the results was carried out to outline the goals attained and the factors limiting implementation of memory aids. After the intervention program, consisting of individual and group activities, VJA showed improvement in level of recall with repetition of tasks, but still required constant external monitoring. Longitudinal follow-up is necessary to obtain more consistent results.

## Abstract (pt)

O termo amnésia refere-se a um estado mental patológico em que a memória e o aprendizado estão afetados em proporções maiores do que as demais funções cognitivas em um paciente sem alteração do nível de consciência. O estudo vigente buscou descrever um caso de amnésia grave em paciente durante reabilitação neurológica e relatar a importância de funções cognitivas preservadas para compensação do *deficit* mnemônico. V.J.A. apresenta quadro clínico sugestivo de Encefalopatia por desnutrição calórico-proteíca, em virtude de vários procedimentos cirúrgicos abdominais em razão de coledocolitíase complicada. Foi realizada análise descritiva dos resultados, buscando detalhar as metas alcançadas e os limitadores para implementação dos auxílios de memória. Após o programa de intervenção, constituído por atividades individuais e grupais (inseridas no contexto e significativas para o paciente), foi observado que V.J.A. apresentou melhora do padrão de evocação com a repetição das tarefas, porém ainda com necessidade de monitoração externa constante. Faz-se, então, necessário acompanhamento longitudinal para obtenção de resultados mais consistentes.

## INTRODUCTION

There are different types of amnesia, but the most commonly studied is the amnestic syndrome, also called global amnesia, characterized by impaired ability to retain new information after brain damage, not restricted to any sensory modality or type of specific material.^1,2^ Anterograde amnesia is often, but not always, accompanied by a deficit in remembering facts and events acquired during the pre-morbid period. The deficits seen in the amnestic syndrome are selective, affecting declarative or explicit memory, while preserving implicit and short-term memory.^3-6^ Some cognitive functions, such as attention, are important for information processing, since attention is a step in the memorization process, and attentional interventions should be performed in patients with amnesia.^7^

The etiology of organic amnesia is highly varied, and whose cause can range from cranial trauma to nutritional deficiency.^3^ Thiamin deficiency can lead to damage or death of neurons, particularly in the thalamus and mammillary bodies. The mammillary bodies receive many neural connections from the hippocampus, which appears to be the primary part of the brain involved in the formation of memories Mammillary body neurons project to the thalamus, which in turn makes connections with the cerebral cortex, where long-term memories are stored. These anatomo-functional correlations may explain why lesions involving the thalamus and mammillary bodies can lead to anterograde amnesia.^8,9^

Thiamine deficiency in the absence of an alcohol-use disorder can cause the full clinical spectrum of Wernicke-Korsakoff syndrome.^10^ The aim of this study, therefore, was to report a case of global amnesia due to nutritional deficiency in an internal neurological rehabilitation program.

## PARTICIPANT

We report the case of VJA, a 57-year-old, married man, with sixth grade elementary school education, and occupation of farmer. He presented cognitive alterations after several abdominal surgical procedures due to complicated choledocholithiasis in June 2014. The patient was fed a parenteral diet for 15 days. The patient was admitted into the rehabilitation program on 07/07/2016. He had lost about 20kg at this time. On the third postoperative day, the patient exhibited psychomotor agitation, visual hallucinations, delusions and alteration of the sleep-wake cycle. He received vitamin B12, where dose and duration of the treatment were not known. No reference was made to thiamine supplementation. Upon admission to the neurological rehabilitation program, laboratory tests, which included determinations of vitamin D3 and vitamin B12 levels, revealed only vitamin D3 insufficiency. Magnetic resonance imaging of the brain showed discrete changes in signals in the subcortical white frontoparietal matter on the left, with a non-specific aspect to the method.

**Procedures.** The neuropsychological assessment was performed using a flexible battery, in 2 sessions lasting approximately 1 hour 30 minutes each. Tests were applied to assess cognitive functions (attention, memory, visuo-constructive and executive functions) and included Information, Mental Control and Logical Memory,^11^ Verbal fluency,^12^ Learning, Naming, Praxias and Viso-construction,^13^ Visual attention,^14^ Comprehension and Digits Span subtests;^15^ Besides qualitative evaluation of communicative ability, reading, writing and calculus abilities were assessed. The neuropsychiatric inventory was used to evaluate behavioral aspects.^16^

The intervention entailed 12 sessions of 60 minutes each given over a one-month period of the program. Two months after the sessions, the patient was admitted to the rehabilitation program again for reinforcement of the guidance over a two-week period. Clinical data (etiology, time of injury, medications, dosage and times of use) and socio-demographic data were obtained from an electronic medical record. The patient provided written informed consent for the study, approved by the Sarah Network Ethics Committee of Rehabilitation Hospitals.

**Data analysis.** A descriptive analysis of the results was performed. Continuous data were obtained using cognitive tests and a behavioral scale. Neuropsychological reassessment was not performed with the same baseline protocol, due to the short interval (period of less than 6 months), in order to avoid the learning effect or bias in the results. In view of this aspect, an ecological reevaluation was carried out, observing the patient's performance on the activities of the rehabilitation program.

## RESULTS

Before the neurological condition, VJA had worked in the fields (plowing and planting). According to his wife, since the neurological event VJA became quieter and more introspective, presenting episodes of crying when talking about the death of parents. He experienced the suffering as if it were current, failing to perform the mnemonic judgment that the event had occurred some years before the neurological injury. However, he did not exhibit behavioral changes, such as irritability or nervousness.

Neuropsychological evaluation was performed. Cognitive impairment was evidenced on memory-related tests. The patient could not retain nor recall information, regardless of the type of material. Immediate memory was preserved, with less impairment on span tests and recency effects in word lists. In addition to memory, VJA had visuo-constructive and planning deficits, albeit less severe than the mnemonic deficit (Table 1). The set of findings was compatible with the diagnosis of WKS. No changes were found on structural imaging, e.g. lesions involving diencephalic structures such as the dorsomedial nucleus of the thalamus or mammillary bodies. Considering that the condition had occurred approximately two years earlier, there was no indication of thiamine replacement.

**Table 1:** Raw scores on cognitive function evaluation tests (attention, memory, visuo-construction and executive functions).

Cognitive Testing	Raw Score
Guidance and information (WMS)	0
Mental Control (WMS)	4/6
Logical memory (WMS)	Immediate: 7/50 Delayed: 0/50
Verbal learning (BEC 96)	10/16
Appointment (BEC 96)	12/12
Verbal fluency (Animals)	5
Visuo-construction (BEC 96)	7/12
Visuo-graphic Attention (BEC 96)	No difficulty
Digit Span (WAIS-III)	Forward: 5 Backward: 3
Trail Making Test	A: 1 error B: > 3 errors (Did not remember the test set)
Clock Drawing	No recall
Recall of figures (BEC 96)	Spontaneous: 0/6 Recognition: 5/6
Calculations (WAIS-III)	16/22
Reading	No difficulty
Writing	No difficulty

The patient was submitted to a neuropsychological intervention, whose initial goals were: to improve temporal orientation and attention, and also to increase interpersonal contact. Goals established later were: to include compensatory memory strategies and introduce meaningful activities to assist transfer to the daily context and facilitate the process of memory and learning. The interventions were structured, individually and in groups, entailing daily activities from 8am-5pm for a period of 1 month. Throughout the rehabilitation program, the daily and systematic use of the calendar after breakfast was worked out, a weekly activity table was drawn up, and importance of the appointment book emphasized; clues and tips were provided to aid recall of people's names and photographs used to recall recent and recent events. VJA had a significant memory deficit, but the other cognitive functions were generally preserved. The use of the clues, in a repetitive and contextualized form, facilitated learning and promoted greater autonomy in his daily routine. The wife was then instructed to be a facilitator and mediator in this process, comprising the main aspect in this rehabilitation.

The group intervention was aimed at stimulating attentional and interpersonal aspects through games and workshops that were part of the patient's daily life, i.e. , training in a more functional and contextualized way. The one-hour game workshops sought to train attention and, consequently, the retention and recall of information through repetition, since attentional ability can facilitate in the mnemonic process and learning. The 2-hour workshop also had the objective of verbal learning, using MDF material for the construction, painting and decoration of a tray, pencil holder and box. The culinary workshop was devoted to the preparation and execution of recipes together with other patients. VJA was motivated to give an opinion on all the proposed interventions, aiming to get him involved as much as possible, in a more meaningful, pleasant and contextualized way, in his daily routine. Thus, the intervention in the attentional function was performed considering generalization to functional activities in the same routine (Table 2).

**Table 2:** Structuring of care (individual and group modality).

Individual intervention	Group intervention
Compensatory Strategies	Functional cognitive training, contextualized
Using calendar and annotations of prospective appointments	Games Workshop
Preparation of workbook	Office joinery, paintingCooking Activity
Training and Exercise	
Reminiscence Technique	
Session with photos of relatives to remember events	
Faded Tips	
Learning without error	
Psychoeducation	
Clarification to wife about cognitive functioning, in particular, the mnemonic alterations and their day-to-day management	

VJA needed external monitoring to remember the activities. With repetition, there was an improvement in retention capacity and recall of information. The memory difficulty required a slow, structured approach, making it necessary to always revisit the themes worked on in the previous session and to help him maintain a sequence of ideas to elaborate in the initiated questions.

The wife's care was fundamental in ensuring implementation of compensatory strategies, as VJA needed mediation to remind him to carry out the activities until he had learned through repetition of the same sequence of activities daily.

## CONCLUSION

Patients with this type of neuropsychological condition need external monitoring to implement the strategies and for generalization to activities.^16-20^ VJA had residual ability, but significant memory deficits affected its functioning.^21^ The memory changes reflected retention difficulties, as there was no benefit in providing clues to recall information. During the rehabilitation program, however, he showed slight improvement in information retention capacity, but still needed to use clues and strategies for recall of the material.

This study had, as limitations, insufficient time to perform the neuropsychological reassessment (with the same baseline protocol) to measure the benefits of the intervention in the case of severe global amnesia. Intensity of follow-up was also lacking, but the family members are instructed to continue their daily routine in a contextualized way (principle of generalization).
